# Chronic Intestinal Failure During the Neonatal Period Related to Height at Five Years of Age

**DOI:** 10.1111/apa.70561

**Published:** 2026-04-28

**Authors:** Johanna Mårtenson, Helena Borg, Julius Kristjansson, Anton Holmgren, Nils Ekvall, Anders Elfvin

**Affiliations:** ^1^ Department of Pediatrics Institute of Clinical Sciences, Sahlgrenska Academy, University of Gothenburg Gothenburg Sweden; ^2^ Department of Pediatrics Sahlgrenska University Hospital, Region Västra Götaland Gothenburg Sweden; ^3^ Department of Pediatric Surgery Sahlgrenska University Hospital, Region Västra Götaland Gothenburg Sweden; ^4^ Department of Pediatrics Halland Hospital Halmstad Sweden; ^5^ Department of Research and Development Region Halland Halmstad Sweden

**Keywords:** chronic intestinal failure, growth, height, Necrotising enterocolitis, neonatology

## Abstract

**Aim:**

To clarify if children with chronic intestinal failure during the neonatal period had a different height at 5 years of age compared to standardised Swedish growth charts.

**Methods:**

This retrospective cohort study of children with chronic intestinal failure during the neonatal period in Gothenburg between 2004 and 2018. Inclusion criterion was parenteral nutrition > 60 days with onset within 3 months from term gestational age. For each child born before week 28 + 0, a control child was identified.

**Results:**

Of the 57 children included, 83% were shorter than the population average, and 12% had a height below −2 standard deviations (SD) at 5 years of age, according to the standard growth chart. The height *Z*‐score (standard deviation score) was significantly lower than that of the reference population; median −1.1 SD (range −4.0–2.1). Children with chronic intestinal failure, born before week 28 + 0, were not significantly shorter than their matched controls.

**Conclusions:**

The majority (88%) of children with chronic intestinal failure had a height growth within the normal reference range, even though they had a shorter mean height. These findings suggest that current treatment strategies support overall satisfactory growth outcomes, although further refinement may be required to promote optimal growth.

## Key Notes

- Children with chronic intestinal failure during the neonatal period had a shorter mean height at 5 years of age compared to standardised normal Swedish growth charts.
- The majority of the children with chronic intestinal failure, 83%, had a height below 0 SD, but only 12% below the normal reference range of −2 SD at 5 years of age.
- The most common cause of chronic intestinal failure among the included children was necrotising enterocolitis.

**Abbreviations**
Chronic IF: chronic intestinal failure
ENS: enteric nervous system
GTQ: gastroschisis
HD: hirschsprung's disease
NEC: necrotising enterocolitis
PIPO: paediatric intestinal pseudo‐obstruction
PN: parenteral nutrition
SBS: short bowel syndrome
SD: standard deviation
SIA: small intestinal atresia
SNQ: swedish neonatal quality register

## 1 Introduction

The characterisation of chronic intestinal failure (chronic IF) is a clinical gastrointestinal condition where the nutritional and/or fluid needs of the gastrointestinal tract cannot be met with the current bowel function. Chronic IF is associated with increased mortality and morbidity and parenteral nutrition has a crucial role for survival in this patient group [1].

In children, the most common cause of chronic IF is short bowel syndrome (SBS), usually with onset after bowel surgery in the neonatal period. Common causes of bowel surgery may be a congenital disease such as gastroschisis (GTQ) or small intestinal atresia (SIA) or an acquired disease such as necrotising enterocolitis (NEC) or midgut volvulus [1]. SBS occurs when patients lose a significant length of the small bowel leading to malabsorption [2]. The incidence overall of SBS is 24.5 per 100 000 live births, with a higher incidence among preterm infants [3].

NEC is a severe gastrointestinal condition mostly affecting preterm infants [4]. It is associated with a high mortality rate, and among survivors there is a risk of significant long‐term sequelae [5]. Surgical treatment of NEC can result in SBS and chronic IF. Medically treated NEC is also related to an increased risk of chronic IF, presumably related to inflammatory and ischaemic injury to the intestine [6].

Growth serves as an indicator of health in the paediatric population, and deviations from normal growth patterns may signal underlying medical conditions or psychosocial challenges [7]. The resemblance in height among family members implies that 80% of the variation in height is subject to genetic influence, while the remaining portion is influenced by environmental factors like diet and exposure to illness [8, 9]. Infants born extremely preterm, before week 28 + 0, are at high risk of poor growth development related to preterm birth [10].

If a child undergoes a period of impaired linear growth due to a medical condition, subsequent catch‐up growth may occur, allowing growth to return to the expected trajectory [11]. The normal reference range for height, weight or head circumference is the mean +/− 2 SD and values outside this range should be considered possible markers of illness, disease or syndrome [12].

There is a need for additional research about the correlation between chronic IF during the neonatal period and height growth at five years of age. It is known that growth regarding these children often is poor because of overestimation of the enteral absorption and inadequate total intake of energy and protein through enteral and parenteral nutrition [1].

The aim of this study was to clarify if children with chronic IF during the neonatal period had a different mean height at five years of age compared to standardised normal Swedish growth charts. A secondary aim was to study if children born before gestational week 28 + 0, with chronic IF during the neonatal period, had poorer height growth at five years of age compared to matched controls.

## 2 Patients and Methods

### 2.1 Ethics and Consent

Ethical approval was obtained from the Swedish ethical review authority. Dnr 2022–04273‐0 and Dnr 2023–05151‐01.

### 2.2 Study Design

This was a retrospective cohort study of children born between 1st January 2004 and 31st December 2018 and treated at Queen Silvia Children's Hospital in Gothenburg. Fifty‐seven children with chronic IF during the neonatal period who met our inclusion criteria were included. The primary outcome was height at five years of age. The height and weight measurements closest to five years of age were used and extrapolated to represent exact five years of age.

### 2.3 Definitions

Chronic IF during the neonatal period was defined as the need for total parenteral nutrition (PN) or partial PN for more than 60 days in total and onset of IF within three months from birth in term infants, and three months corrected gestational age in preterm infants.

### 2.4 Subjects

The study subjects were collected from the local register of the IF Centre of Gothenburg, the paediatric surgical unit and the neonatal intensive care unit of Gothenburg. Information on demographics, diagnoses and medical history was abstracted. The aim of the study was to include as many subjects as possible with chronic neonatal IF; however, we do not claim to have identified all IF patients during the study period, and therefore the study does not make any calculations regarding incidence or prevalence of neonatal IF during the studied years.

For every child born before gestational week 28 + 0 a control child who had also been admitted to the neonatal intensive care unit of Gothenburg was identified. The control child was matched to the chronic IF case by sex and gestational age at birth. The cases and controls were matched in pairs but analysed on group level. As infants born before 28 + 0 weeks' gestation are at high risk of impaired growth due to preterm birth, we considered it relevant to compare those with chronic intestinal failure (chronic IF) to a control group, in order to evaluate chronic IF as an independent factor influencing growth. Control infants were excluded if they had necrotising enterocolitis (NEC), had undergone bowel surgery, or presented with any congenital syndrome. Chronic IF children born before gestational week 28 + 0 were compared both to the matched control group and to the standardised normal Swedish growth chart. Data for the whole group of chronic IF patients were not corrected for gestational age, nor for the underlying condition causing the intestinal failure.

### 2.5 Inclusion and Exclusion Criteria

**FIGURE 1:**
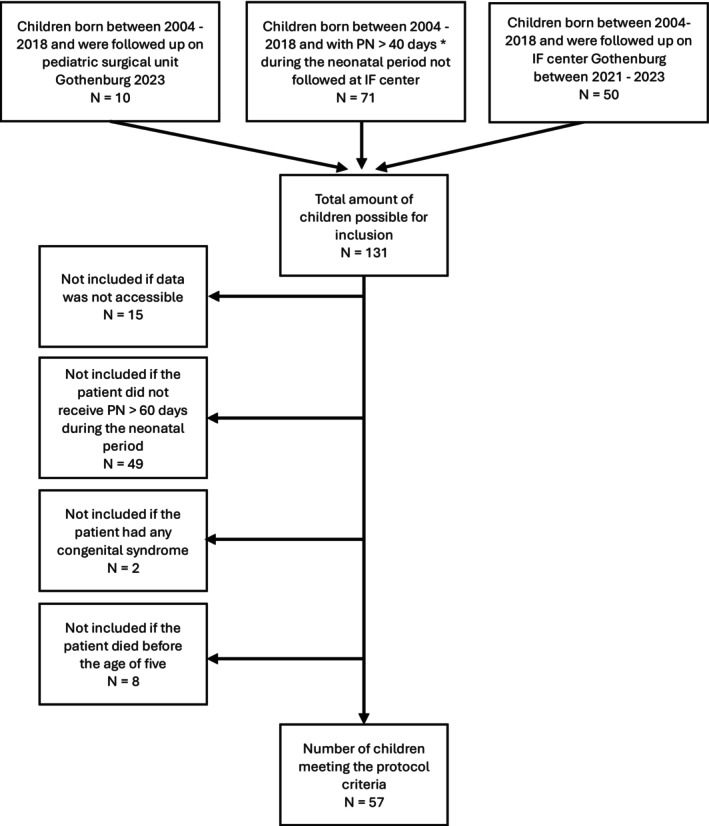
Flowchart of children included in the study with chronic IF during the neonatal period in Gothenburg 1 January 2004–31 December 2018. PN, parenteral nutrition; SNQ, swedish neonatal quality register; N, number; Chronic IF, chronic intestinal failure; * All children with PN > 40 days analysed, not to miss any with PN > 60 days. Children with PN between 40 to 59 days were then excluded from further analysis. Chronic IF was defined as PN > 60 days.

Children born after 2004 with chronic IF during the neonatal period, who were at least five years old at the time of the study, were included. Not to miss any cases, all children with PN > 40 days were evaluated to check the exact number of days with PN and excluded if the number of days with PN was < 60 days (Figure 1). Mortality before the age of five and congenital syndrome were exclusion criteria. Children with absence of, or incomplete medical records were not included.

Data were analysed using IBM SPSS (Statistical Package for the Social Sciences) version 29. Frequencies and percentages were used for categorical variables. Medians and full range were used for continuous variables. All continuous variables have also been calculated as mean (± standard deviation), but only the median (range) is presented.

To compare categorical variables, Chi‐square or Fishers exact test were used. To compare numeric variables, an independent *t*‐test was used for normally distributed data, and Mann–Whitney *U*‐test was used if not normally distributed. Standard deviation score (SDS) and the term *Z* score may be used interchangeably, as they are in this article. We have calculated *Z* scores (SDS) on height and weight. Both the original value and the *Z* score on height and weight were compared to the average at the age of five years according to the Swedish growth chart, using one‐sample *t*‐test [12]. The statistical *p*‐value has been calculated for the groups both on the height (cm), weight (kg), and the *Z* score (SD).

A power calculation was performed for the primary research question using one study group compared to the normal growth chart developed by Albertsson–Wikland et al. The growth chart value for height at five years of age was used as the fixed value, and the mean height growth for the chronic IF group was used as the study variable. With a *p* < 0.05 and a power of 80%, a total of 50 children with chronic IF had to be included if assuming that children with chronic IF are on average 2 cm shorter at five years of age compared to the Swedish reference [12].

The *Z* score (SDS) was calculated using age and sex specific mean and SD based on the standard growth curves presented by Albertsson Wikland et al. 2002 [12]. An algorithm with perfect fit to the reference at that age has been created by dr Niklasson, one of the co‐authors of the Swedish reference from 2002, so that SDS can be calculated at any age in the actual age span. SDS was calculated exactly as *Z* score but adjusted for age and sex according to the reference used.

The *Z* score (SDS) was calculated from the actual age at measurement. Data on the exact height at five years of age could not be found among all patients, and to make data more accessible, data on height at the time of measurement as close as possible to the age of five was collected and extrapolated to exact five years of age. The mathematical extrapolation is exact, but the uncertainty depends on if the child grows in accordance with the curve from the time of measurements to 5 years of age [12].

## 3 Results

### 3.1 Descriptive Results

**FIGURE 2:**
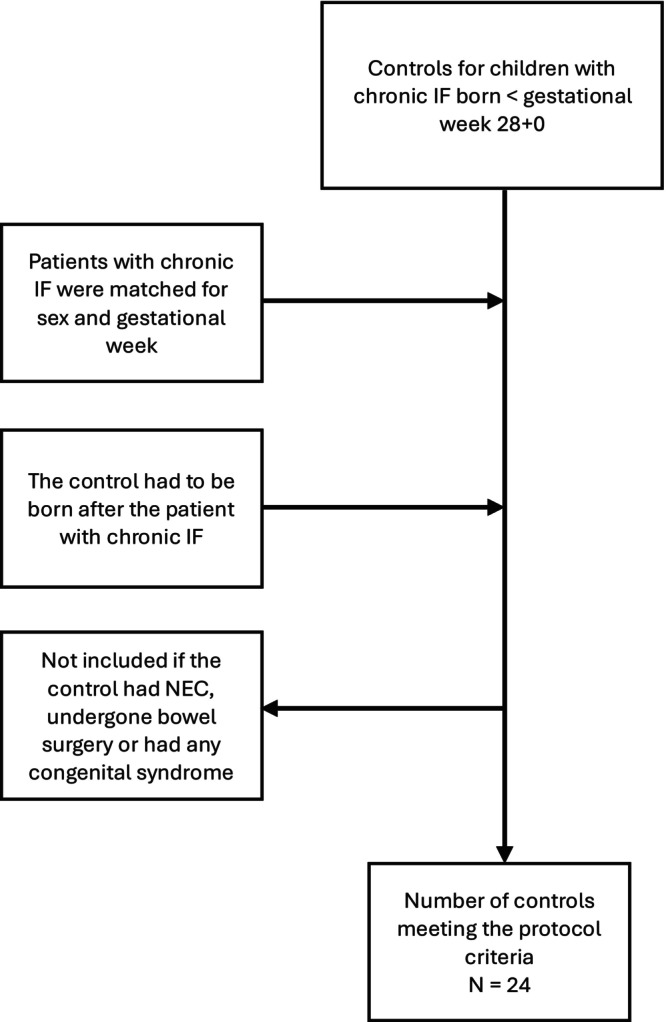
Flowchart of included controls for children with chronic IF born gestational week 28 + 0 in Gothenburg 1 January 2004–31 December 2018. Chronic IF, chronic intestinal failure; N, number.

Between January 1st of 2004 and December 31st 2018 a total of 131 children were analysed for possible inclusion in this study. Of these 131 children, 71 had PN more than 40 days during the neonatal period and were registered in the SNQ, 50 children were followed up by the IF centre in Gothenburg between 2021 and 2023 and 10 were followed up on paediatric surgical unit in Gothenburg. Fifty‐seven of the 131 children met the inclusion criteria (Figure 1). Of the 57 included children, 24 were born before gestational week 28, and were matched to controls (Figure 2). Regarding all 57 chronic IF patients, 41 were born before gestational week 37, with a median age at birth of 28 w.

Presented in Table 1 are the characteristics of children with chronic IF during the neonatal period admitted to the Neonatal intensive care unit or the IF centre at Queen Silvia Children's Hospital in Gothenburg during the study period. Diagnoses associated with prematurity are presented in Table 1, with no differences between the cases and controls born before week 28 + 0. Birth data regarding the controls born before week 28 + 0 are presented in Table 1.

**TABLE 1:** Characteristics of children with chronic IF during the neonatal period admitted to the Neonatal intensive care unit at Queen Silvia Children's Hospital in Gothenburg and IF center of Gothenburg between January 1st 2004 to December 31st 2018.

Infant characteristics	All Children (*n* = 57)	Children followed at the IF center (*n* = 21)	Children born ≥ week 28 + 0 not followed at the IF center (*n* = 12)	Children with chronic IF born < week 28 + 0 not followed at IF center (*n* = 24)	Non‐IF Controls for children born < week 28 + 0 (*n* = 24)	P‐value of children with chronic IF born < w. 28 + 0 compared to controls
Gestational age at birth, weeks	28 (22; 42)	37 (29; 42)	32 (28; 39)	25 (22; 27)	25 (22; 27)	ns
Female, sex, *n*	27 (47%)	9 (43%)	5 (42%)	13 (54%)	13 (54%)	ns
Length at birth, cm	32 (27; 56)	50 (44; 56)	39 (32; 52)	31 (27; 37)	31 (27; 38)	0.748
Weight at birth, g	1110 (440; 4560)	3475 (1716; 4560)	1658 (740; 3540)	678 (440; 1110)	698 (420; 1060)	0.951
Head circumference at birth, cm	25 (19; 37)	36 (31; 37)	28 (26; 36)	23 (19; 27)	23 (19; 27)	0.861
Days with PN				80 (60; 279)	17 (0; 45)	< 0.001
PN at five years of age	17 (30%)	17 (81%)	0 (0%)	0 (0%)	0 (0%)	ns
History of any bowel surgery	53 (93%)	20 (95%)	12 (100%)	21 (88%)	0 (0%)	< 0.001
History of any intestinal resection	48 (84%)	16 (76%)	12 (100%)	20 (83%)	0 (0%)	< 0.001
Medical diagnoses associated with chronic IF*						
NEC	17 (30%)	1 (5%)	2 (17%)	14 (58%)	0 (0%)	
GTQ	8 (14%)	4 (19%)	4 (33%)	0 (0%)	0 (0%)	
SIA	4 (7%)	4 (19%)	0 (0%)	0 (0%)	0 (0%)	
Volvulus	6 (11%)	3 (14%)	2 (17%)	1 (4%)	0 (0%)	
PIPO	3 (5%)	3 (14%)	0 (0%)	0 (0%)	0 (0%)	
HD	6 (11%)	5 (24%)	1 (8%)	0 (0%)	0 (0%)	
Congenita enteropathies	1 (2%)	1 (5%)	0 (0%)	0 (0%)	0 (0%)	
Other	15 (26%)	3 (14%)	3 (25%)	9 (38%)	0 (0%)	
PDA, surgical treatment, *n* **			3 (25%)	13 (54%)	12 (50%)	1.000
PDA, medical treatment, *n*			1 (8%)	12 (50%)	10 (42%)	0.772
Any BPD			1 (8%)	17 (71%)	18 (75%)	1.000
IVH grade 3–4			0 (0%)	5 (21%)	2 (8%)	0.416
ROP stages 3–5			1 (8%)	16 (67%)	10 (42%)	0.234

*Note:* Median (min; max) for continuous variables, number (%) for categorical variables. Other diagnoses associated with chronic IF include: meconium ileus, obstructive ileus due to milk curd syndrome, ileus, prematurity, intestinal malfunction due to sepsis or suspected NEC, oesophageal atresia with tracheoesophageal fistulae and cloacal malformation.

Abbreviations: BPD, bronchopulmonary dysplasia; Chronic IF, chronic intestinal failure; GTQ, gastroschisis; HD, Hirschsprung's disease; IVH, intraventricular haemorrhage; NEC, necrotising enterocolitis; ns, no significance; PDA, patent ductus arteriosus; PIPO, paediatric intestinal pseudo‐obstruction; PN, parenteral nutrition; ROP, retinopathy of prematuritySIA, small intestinal atresia.

* *The total exceeds 100%, since some children may have received more than one diagnosis*.

** *Patient can be included in both surgical and medical group*.

NEC was the most common cause of chronic IF among the children born before week 28 + 0 (Table 1). All these children had achieved intestinal autonomy and were on full enteral feeds at the age of five years (Table 1). Regarding children identified via the IF centre of Gothenburg HD, SIA and GTQ were the most common diagnosis.

### 3.2 Growth at Five Years of Age

**FIGURE 3:**
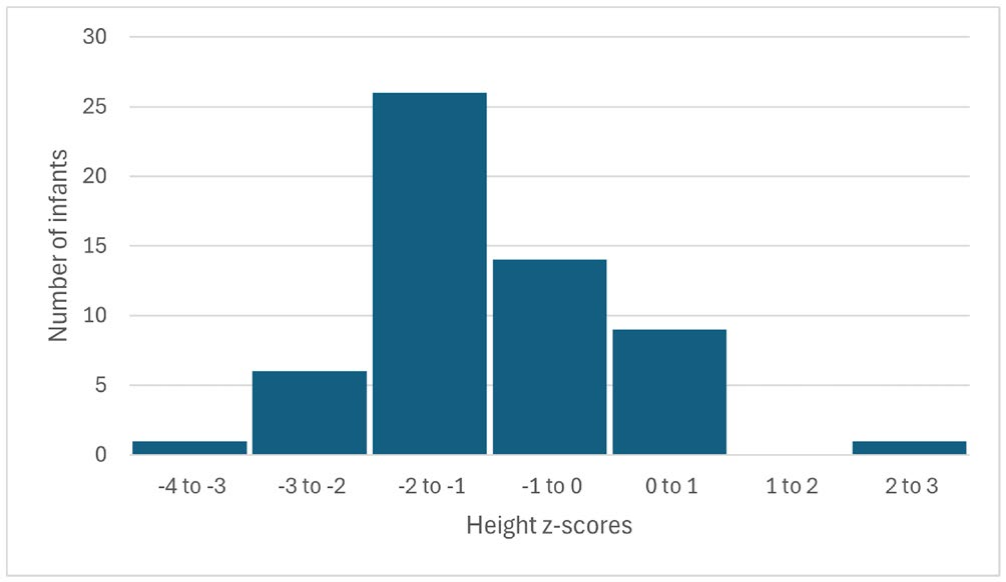
Histogram of height *Z* scores (SDS) at five years of age for the studied cohort of children with intestinal failure.

As shown in Table 2 the mean height and weight of included children were significantly lower (*p* < 0.001) than the mean height and weight of the standardised Swedish growth chart. In addition, when analysed separately, children with chronic IF born before gestational week 28 + 0 had significantly lower mean height and weight at five years of age compared with the standardised Swedish growth chart (Table 2). Of all included children, 83% had a height *Z* score below 0 SD. However, 12% had a height *Z* score below −2 SD, which is often related to as the lower cut off for normal growth (Figure 3).

**TABLE 2:** Growth at five years of age.

Growth at five years of age	All Children (*n* = 57)	*p* *	Children followed at the IF centre (*n* = 21)	*p* *	Children born ≥ week 28 + 0 not followed at the IF centre (*n* = 12)	*p* *	Children born < week 28 + 0 not followed at the IF centre (*n* = 24)	*p* *	Non‐IF Controls for children born < week 28 + 0 (*n* = 24)	*p*s of comparing IF patients born < w. 28 + 0 to controls
Height cm, median (Range)	106 (93; 121)	< 0.001	106 (98; 121)	< 0.001	105 (93; 115)	0.022	106 (100; 113)	< 0.001	109 (96; 119)	0.578
median height *Z* score (Range)	−1.1 (−4.0; 2.1)	< 0.001	−1.3 (−2.9; 2.1)	< 0.001	−1.3 (−4.0; 0.9)	0.019	−1.0 (−2.4; 0.3)	< 0.001	−0.5 (−3.6; 1.8)	0.592
Weight kg, median (Range)	17 (12; 24)	< 0.001	17 (12; 22)	0.014	17 (13; 24)	0.041	17 (13; 21)	< 0.001	17 (12; 25)	0.702
median weight *Z* score (Range)	−1.2 (−3.4; 1.6)	< 0.001	−1.1 (−3.4; 1.1)	0.001	−1.4 (−3.2; 1.6)	0.009	−1.2 (−3.2; 0.7)	< 0.001	−1.2 (−4.2; 2.1)	0.670
Height z‐score below 0 SD *n*(%)	47 (83%)		18 (86%)		8 (67%)		21 (88%)		16 (67%)	
Height *Z* score below −2 SD *n*(%)	7 (12%)		3 (14%)		2 (17%)		2 (8%)		7 (29%)	

Abbreviations: IF, intestinal failure; SD, standard deviation.

* *p‐*value was calculated with a one‐sample *t*‐test by comparing the children in each group to the standardised normal Swedish growth charts.

The median age at time of height measurement was 5.0 years (interquartile range 4.7–5.3 years).

### 3.3 Growth of Chronic Intestinal Failure Patients Born Before Gestational Week 28 + 0, in Relation to Matched Controls

Children with chronic IF, born before gestational week 28 + 0, had a shorter median height *Z* score than their matched controls at five years of age (Table 2). In average the median height differed 3 cm between the groups. However, this difference did not reach statistical significance (*p* = 0.592). Nor was there a significant difference in weight *Z* score between the groups (*p* = 0.670).

### 3.4 Growth of Different Diagnostic Groups of Chronic IF

The large group of children with NEC as the cause of chronic IF was compared to the standardised Swedish growth chart. All other groups combined were then compared to the reference values of the standardised Swedish growth charts.

Regarding children born preterm, both the NEC IF group and the No‐NEC IF group had a height at five years of age significantly below the reference values. However, when comparing the two groups to each other there was no difference in height between the groups at five years of age (*p* = 0.727).

The bowel anatomy characteristics for the different groups of children as presented in Table 3 shows that a majority of all children (75%) had their ileocecal valve (ICV) preserved. Among children born preterm, the ICV was preserved in more than 95% of the cases. Among children followed at the IF centre, around 38% had their ICV remaining (Table 3). Of the 14 children with the ICV removed, 13/14 belonged to the children followed at the Gothenburg IF centre (Table 3). Regarding the small bowel, a few had 100% small bowel remaining, whereas others had only 10% small bowel remaining (Table 3). Of the patients followed at the IF centre, 81% were PN dependent at the age of five years compared to 0% PN dependent at five years of age in the other groups (Table 2).

**TABLE 3:** Bowel anatomy characteristics.

Bowel anatomy	All Children (*n* = 57)	Children followed at the IF centre (*n* = 21)	Children born ≥ week 28 + 0 not followed at the IF centre (*n* = 12)	Children born < week 28 + 0 not followed at IF centre (*n* = 24)
Ileocecal valve preserved	43 (75%)	8 (38%)	12 (100%)	23 (96%)
Ileum preserved	15 (26%)	6 (29%)	3 (25%)	6 (25%)
Jejunum preserved	46 (81%)	13 (62%)	10 (83%)	23 (96%)
Remaining small intestine %	86% (10, 100)	50% (10, 100)	91% (30, 100)	85% (46, 100)
Total colon preserved	39 (68%)	7 (33%)	11 (92%)	21 (88%)
Right colon preserved	42 (74%)	8 (38%)	12 (100%)	22 (92%)
Left colon preserved	46 (81%)	12 (57%)	11 (92%)	23 (96%)
Colon transversum preserved	45 (79%)	9 (43%)	12 (100%)	24 (100%)
Intestinal transplantation	2 (4%)	2 (10%)	0 (0%)	0 (0%)

## 4 Discussion

This retrospective cohort study of 57 children with chronic IF during the neonatal period showed a significantly shorter mean height at five years of age compared to standardised Swedish growth charts for their sex, where 83% of the children with chronic IF during the neonatal period were below the mean height for their sex at five years of age, but the majority (88%) were still within the normal reference range of −2 SD. This implicates that the current treatment strategies for children with chronic IF at the Sahlgrenska University hospital are well‐functioning; however, there is room to improve the growth in this group of children. A majority of the 57 children with chronic IF were born preterm, before 37 gestational weeks. It was not possible in the present study to correct for preterm birth when comparing the data from the IF patients against standardised Swedish growth charts. Trying to evaluate if the shorter height at five years of age could be explained by IF alone or also affected by preterm birth, we compared all children with IF born before gestational week 28 + 0 to matched controls.

The height of children with chronic IF during the neonatal period, born before gestational week 28 + 0, did not differ significantly from their matched controls at five years of age. This implies that children born extremely preterm may grow within the lower normal range and that chronic IF, when adequately managed, does not necessarily further exacerbate impaired linear growth.

Height growth did not differ between different diagnostic groups of chronic IF when compared to standardised Swedish growth charts. It was not possible in the present study to clarify to what degree the underlying condition causing IF influenced height growth. There was no significant difference in the height *Z* score between children who had NEC as the cause of chronic IF compared to the combined group of those who did not have NEC as the cause of chronic IF. Despite that NEC was a transient cause of IF, children with NEC did not grow significantly better than children with other, more persistent causes of chronic IF. However, the majority of the NEC children were born preterm.

The present study suggested that the most common cause of neonatal chronic IF was NEC (30%). Almost all of these patients were born before gestational week 28 + 0. NEC resulted in transient IF and by the age of five years, all but one of these children had regained intestinal autonomy.

In line with the results from the present study, a previous study by Mutanen et al. showed that among children with paediatric SBS‐IF the majority had a height within the normal reference range [13]. Norsa et al. found that long‐term growth in children with neonatal very‐SBS was affected since there was a significant difference between final height and calculated target height [13]. This corresponds to the findings of the present study, where children with chronic IF during the neonatal period had a shorter mean height compared to standardised Swedish growth charts for their sex by the age of five.

A study by Roggero et al. described the growth patterns of 23 infants with IF during their first two years of life. They illustrated a critical phase during the hospitalisation with postnatal growth retardation but also demonstrated catch‐up growth between discharge and 24 months regardless of PN dependency [14]. Their findings were consistent with the results found by McLaughlin et al. who described growth retardation in 41 infants with SBS during the first six months and thereafter increased growth, proceeding into a stabilization of the growth pattern [15]. Moreover, a recently published study by Ali et al. showed that children with IF diagnosed during infancy were shorter than expected for their age. They described a median (min, max) height‐for‐age *Z* score of −1.4 (−4.8, 2.1) and weight‐for‐age *Z* score of −0.6 (−2.7, 1.6) at the age of five [16]. These results correspond to the results of the present study. This may suggest that children with chronic IF remained consistently below average in height and weight and yet stayed within the normal reference range at both two and five years of age. Furthermore, they appeared to follow their own growth trajectory and achieved a stable growth pattern.

When children born before gestational week 28 + 0 were compared to matched controls, there was no significant difference in height at age five years between these groups. This contradicts what a recently published study from our research group has shown, where preterm children diagnosed with NEC during the neonatal period had lower height and weight at five years of age than their matched controls [17]. However, another study of children compared those with NEC, mainly medically treated, to matched controls and found that there was no significant difference in height between the groups at adolescence, which is consistent with our study [18]. The results of the present study indicate that regardless of chronic IF, preterm infants had a height within the lower normal range at five years of age. Other studies have described preterm infants who had surgery due to NEC to have a poor catch up growth during the first year of life [19]. Mutanen et al. found that height z‐scores more often were below −2 SD in patients who remained dependent on PN support [20]. In the present study none of the infants born before gestational week 28 + 0 were PN dependent at five years of age, and among patients with NEC as the cause of chronic IF, only one, born after gestational week 28 + 0 was PN dependent at age five years. NEC causing transient IF resulting in intestinal autonomy at age five years is also shown in a study by Mutanen et al. who identified that neonates with SBS due to NEC weaned of PN sooner than neonates with SBS due to other causes [21]. Furthermore Fredriksson et al. found that among 105 neonates with IF, where NEC was the most common aetiology, the majority of the children had achieved enteral autonomy by the age of one [22].

In the present study, as in other studies by Fallon et al. and by Sparks et al., NEC was the most common diagnosis associated with chronic IF in neonates [23, 24].

### 4.1 Strengths and Weaknesses

A strength of this study was that 57 children with chronic IF during the neonatal period were included. This is to be considered as a large number given that the condition is relatively rare. A weakness with this study was that our unit had a poor registration of total number of days with PN between 2004 and 2007. There were probably children born before 2008 who would have met the inclusion criteria but could not be found. It is a weakness that the study was a single center study, where only the children followed up in Gothenburg at five years of age could be included. Furthermore, data on height at five years of age could not be found among all children. Data on height at an age as close as possible to the age of five was extrapolated to represent the height at five years of age. Parental height was not collected in the present study and any genetical influence on height was therefore not evaluated.

## 5 Conclusions

In conclusion, this single center retrospective cohort study illustrated that children with chronic IF during the neonatal period had a shorter mean height at five years of age compared to healthy Swedish children. The majority of the children with chronic IF, more than 80%, were shorter than average, but only 12% were below the normal reference range, indicating that the current treatment methods of these children were well‐functioning. Children born before gestational week 28 + 0 and with chronic IF during the neonatal period did not have poorer height growth at five years of age compared to matched controls. The height growth did not differ between different groups of chronic IF when compared to standardised normal Swedish growth charts. The most common cause of chronic IF in all studied children was NEC, mainly in children born before gestational week 28 + 0. By the age of five years, most of the children with chronic IF due to NEC had regained intestinal autonomy. The findings of the present study, that children with chronic IF during the neonatal period were shorter than average but within the normal reference range, could be of great value since it indicates that the current treatment methods of this vulnerable patient group are ensuring sufficient nutritional intake. Furthermore, the study clarifies the importance of careful monitoring. In the future, it will be needed to further study growth among children with chronic IF to better understand the association between failing gut, nutrition and growth and to elucidate where improvements can be made.

## Author Contributions

**Johanna Mårtenson:** writing – original draft, conceptualization, methodology, formal analysis, project administration, data curation, validation, investigation, writing – review and editing. **Julius Kristjansson:** methodology, software, data curation, validation, formal analysis, writing – review and editing. **Anders Elfvin:** conceptualization, methodology, software, data curation, investigation, validation, formal analysis, supervision, funding acquisition, visualization, project administration, resources, writing – review and editing. **Helena Borg:** conceptualization, methodology, validation, supervision, writing – review and editing, resources, data curation. **Nils Ekvall:** methodology, validation, formal analysis, supervision, writing – review and editing.

## Funding

This work was supported by grants from the Swedish state under the agreement between the Swedish government and the county councils, the ALF agreement (ALFGBG‐1006755). Open access funding provided by the University of Gothenburg.

## Conflicts of Interest

The authors declare no conflicts of interest.

## Data Availability

The data that support the findings of this study are available on request from the corresponding author. The data are not publicly available due to privacy or ethical restrictions.
